# Author Correction: Super-regional land-use change and effects on the grassland specialist flora

**DOI:** 10.1038/s41467-019-12091-y

**Published:** 2019-09-19

**Authors:** Alistair G. Auffret, Adam Kimberley, Jan Plue, Emelie Waldén

**Affiliations:** 1 Department of Ecology, Swedish University of Agricultural Sciences, Box 704475007 Uppsala, Sweden; 2 Biogeography and Geomatics, Department of Physical Geography, Stockholm University, 10691 Stockholm, Sweden; 3 Department of Biology, University of York, York, YO10 5DD UK; 4 School for Natural Sciences, Technology and Environmental Studies, Södertörn University, 141 89 Stockholm, Sweden

**Keywords:** Biodiversity, Grassland ecology

Correction to: *Nature Communications* 10.1038/s41467-018-05991-y, published online 27 August 2018.

The original version of this Article contained an error in Fig. 1a, in which the change in landscape heterogeneity was incorrectly plotted instead of the intended variable, the change in open land-cover. The data were described correctly in the “Results” text and figure legend of the original article. This has been corrected in both the PDF and HTML versions of the Article.

