# Supplementary material for: Super-regional land-use change and effects on the grassland specialist flora
Source: Nat Commun. 2018 Aug 27;9:3464. doi: 10.1038/s41467-018-05991-y (PMC6110833; doi:10.1038/s41467-018-05991-y)
Supplement: Supplementary file 1 — Supplementary Information [file 41467_2018_5991_MOESM1_ESM.pdf]

SUPPLEMENTARY INFORMATION

Super-regional land-use change and effects on the grassland specialist flora

Alistair G. Auffret, Adam Kimberley, Jan Plue, Emelie Waldén

Supplementary Table 1. Summary of landscape change in southern Sweden between 1940s-60s and 2016. Fraction change in arable, open and forest cover across the whole study area and the fraction of 6733 landscapes that decreased in heterogeneity across the time period.

	Arable change	Open change	Forest change	Decreased heterogeneity
All landscapes (n= 6733)	-0.19	-0.17	0.11	0.67
Arable landscapes (n=1683)	-0.11	0.58	-0.03	0.34
Open landscapes (n=1683)	-0.29	-0.55	0.38	0.94
Forest landscapes (n=1683)	-0.34	-0.15	0.05	0.72

Supplementary Table 2. Full generalised linear model explaining species richness in 23 398 Swedish semi-natural grasslands including both landscape and local predictor variables plus the county in which the grassland is located, including 95% confidence intervals. All variables were standardised for comparison.

Variable	Estimate	Lower	Upper	Std. Error	t value	Pr(>|t|)
Intercept	2.028	1.988	2.067	0.020	100.526	0.000
Landscape						
Present landscape open	0.024	0.009	0.038	0.007	3.220	0.001
Past landscape heterogeneity	-0.037	-0.049	-0.024	0.006	-5.692	0.000
Past landscape arable	-0.080	-0.094	-0.066	0.007	-11.366	0.000
Local						
Grassland area (log)	0.178	0.162	0.195	0.009	20.863	0.000
Grassland heterogeneity	0.220	0.206	0.234	0.007	30.769	0.000
Grassland open cover	0.069	0.055	0.082	0.007	9.730	0.000
Grassland improvement	-0.111	-0.125	-0.096	0.007	-14.958	0.000
Area Fennoscandian species-rich dry-mesic lowland grassland (log x + 1)	0.254	0.241	0.267	0.007	38.445	0.000
Area Semi-natural dry grassland and shrubland on calcareous substrates (log x + 1)	0.116	0.105	0.128	0.006	19.451	0.000
Region (baseline=Kronoberg)						
Blekinge	-0.526	-0.577	-0.476	0.026	-20.417	0.000
Gotland	-0.052	-0.104	0.000	0.027	-1.957	0.050
Halland	-0.301	-0.351	-0.252	0.025	-11.870	0.000
Jönköping	-0.145	-0.190	-0.101	0.023	-6.389	0.000
Kalmar	-0.013	-0.060	0.033	0.024	-0.563	0.574
Skåne	-0.289	-0.356	-0.223	0.034	-8.473	0.000
Sodermanland	-0.169	-0.234	-0.105	0.033	-5.179	0.000
Stockholm	-0.324	-0.379	-0.268	0.028	-11.482	0.000
Uppsala	0.109	0.067	0.151	0.021	5.097	0.000
Värmland	-0.013	-0.060	0.034	0.024	-0.533	0.594
Västmanland	-0.397	-0.453	-0.342	0.028	-14.042	0.000
Västra Götaland	0.003	-0.043	0.049	0.024	0.108	0.914
Örebro	-0.171	-0.214	-0.128	0.022	-7.763	0.000
Östergötland	-0.149	-0.192	-0.105	0.022	-6.717	0.000

Supplementary Table 3. Generalised linear models using landscape, local or regional variables to explain species richness in 23 398 Swedish semi-natural grasslands, including 95% confidence intervals (three models). All variables were standardised for comparison.

Variable	Estimate	Lower	Upper	Std. Error	t value	Pr(>|t|)
Landscape model						
Intercept	1.945	0.004	1.937	1.953	498.958	0.000
Present landscape open	0.127	0.007	0.112	0.141	17.118	0.000
Past landscape heterogeneity	0.008	0.008	-0.007	0.024	1.065	0.287
Past landscape arable	-0.073	0.008	-0.089	-0.057	-9.130	0.000
Local model						
Intercept	1.910	0.003	1.903	1.917	547.183	0.000
Grassland area (log)	0.188	0.008	0.172	0.205	22.511	0.000
Grassland heterogeneity	0.200	0.007	0.186	0.214	27.345	0.000
Grassland open cover	0.047	0.007	0.033	0.061	6.544	0.000
Grassland improvement	-0.116	0.008	-0.130	-0.101	-15.271	0.000
Area Fennoscandian species-rich dry-mesic lowland grassland (log x + 1)	0.273	0.006	0.260	0.286	42.107	0.000
Area Semi-natural dry grassland and shrubland on calcareous substrates (log x + 1)	0.139	0.006	0.128	0.150	24.145	0.000
Region model (baseline=Kronoberg)						
Intercept	2.099	0.023	2.053	2.144	89.880	0.000
Blekinge	-0.542	0.030	-0.600	-0.483	-18.219	0.000
Gotland	-0.100	0.031	-0.160	-0.039	-3.244	0.001
Halland	-0.432	0.029	-0.489	-0.375	-14.861	0.000
Jönköping	-0.214	0.026	-0.265	-0.162	-8.125	0.000
Kalmar	-0.048	0.028	-0.102	0.007	-1.726	0.084
Skåne	-0.324	0.040	-0.402	-0.247	-8.179	0.000
Sodermanland	-0.321	0.038	-0.395	-0.247	-8.491	0.000
Stockholm	-0.452	0.033	-0.516	-0.389	-13.914	0.000
Uppsala	0.076	0.025	0.027	0.125	3.048	0.002
Värmland	-0.012	0.028	-0.067	0.043	-0.440	0.660
Västmanland	-0.394	0.033	-0.457	-0.330	-12.054	0.000
Västra Götaland	0.022	0.026	-0.029	0.074	0.838	0.402
Örebro	-0.218	0.025	-0.268	-0.168	-8.551	0.000
Östergötland	-0.139	0.025	-0.189	-0.089	-5.504	0.000

Supplementary Table 4. Generalised linear model explaining species richness in 23 398 Swedish semi-natural grasslands including landscape variables plus the county in which the grassland is located, including 95% confidence intervals. All variables were standardised for comparison.

Variable	Estimate	Lower	Upper	Std. Error	t value	Pr(>|t|)
Intercept	2.126	2.081	2.171	0.023	91.987	0.000
Landscape						
Present landscape open	0.143	0.129	0.158	0.008	18.834	0.000
Past landscape heterogeneity	-0.025	-0.040	-0.011	0.008	-3.368	0.001
Past landscape arable	-0.109	-0.125	-0.093	0.008	-13.535	0.000
Region (baseline=Kronoberg)						
Blekinge	-0.576	-0.634	-0.518	0.030	-19.490	0.000
Gotland	-0.101	-0.160	-0.041	0.031	-3.293	0.001
Halland	-0.464	-0.521	-0.408	0.029	-16.062	0.000
Jönköping	-0.241	-0.292	-0.189	0.026	-9.202	0.000
Kalmar	-0.033	-0.086	0.021	0.027	-1.202	0.229
Skåne	-0.303	-0.380	-0.227	0.039	-7.747	0.000
Sodermanland	-0.348	-0.422	-0.275	0.038	-9.276	0.000
Stockholm	-0.485	-0.548	-0.422	0.032	-15.054	0.000
Uppsala	0.066	0.017	0.114	0.025	2.657	0.008
Värmland	-0.003	-0.057	0.051	0.028	-0.124	0.901
Västmanland	-0.448	-0.512	-0.384	0.033	-13.741	0.000
Västra Götaland	-0.080	-0.132	-0.028	0.027	-3.006	0.003
Örebro	-0.237	-0.286	-0.187	0.025	-9.406	0.000
Östergötland	-0.204	-0.253	-0.154	0.025	-8.094	0.000

Supplementary Table 5. Generalised linear model explaining species richness in 23 398 Swedish semi-natural grasslands including both local predictor variables and the county in which the grassland is located, including 95% confidence intervals. All variables were standardised for comparison.

Variable	Estimate	Lower	Upper	Std. Error	t value	Pr(>|t|)
Intercept	2.009	1.969	2.048	0.020	99.498	0.000
Local						
Patch area (log)	0.179	0.162	0.196	0.008	21.164	0.000
Patch heterogeneity	0.226	0.212	0.241	0.007	31.612	0.000
Grassland open cover	0.071	0.057	0.085	0.007	10.128	0.000
Grassland improvement	-0.120	-0.135	-0.106	0.007	-16.336	0.000
Area Fennoscandian species-rich dry-mesic lowland grassland (log x + 1)	0.250	0.237	0.263	0.007	38.135	0.000
Area Semi-natural dry grassland and shrubland on calcareous substrates (log x + 1)	0.120	0.109	0.132	0.006	20.703	0.000
County (baseline=Kronoberg)						
Blekinge	-0.520	-0.571	-0.470	0.026	-20.222	0.000
Gotland	-0.032	-0.084	0.020	0.027	-1.195	0.232
Halland	-0.264	-0.313	-0.214	0.025	-10.411	0.000
Jönköping	-0.113	-0.158	-0.069	0.023	-4.997	0.000
Kalmar	-0.013	-0.059	0.034	0.024	-0.544	0.587
Skåne	-0.298	-0.365	-0.231	0.034	-8.703	0.000
Sodermanland	-0.127	-0.191	-0.064	0.033	-3.904	0.000
Stockholm	-0.297	-0.352	-0.241	0.028	-10.516	0.000
Uppsala	0.124	0.082	0.166	0.021	5.773	0.000
Värmland	-0.015	-0.062	0.032	0.024	-0.624	0.533
Västmanland	-0.348	-0.403	-0.293	0.028	-12.377	0.000
Västra Götaland	0.038	-0.007	0.084	0.023	1.633	0.103
Örebro	-0.162	-0.205	-0.118	0.022	-7.322	0.000
Östergötland	-0.129	-0.172	-0.085	0.022	-5.815	0.000

Supplementary Table 6. Generalised linear model explaining species richness in 23 398 Swedish semi-natural grasslands including landscape and local predictor variables including 95% confidence intervals. All variables were standardized for comparison.

Variable	Estimate	Lower	Upper	Std. Error	t value	Pr(>|t|)
Intercept	1.909	1.902	1.916	0.003	548.739	0.000
Landscape						
Present landscape open	0.002	-0.013	0.016	0.007	0.225	0.822
Past landscape heterogeneity	-0.010	-0.023	0.003	0.007	-1.471	0.141
Past landscape arable	-0.071	-0.085	-0.057	0.007	-10.160	0.000
Local						
Grassland area (log)	0.193	0.176	0.210	0.009	22.620	0.000
Grassland heterogeneity	0.196	0.182	0.210	0.007	26.818	0.000
Grassland open cover	0.050	0.036	0.064	0.007	6.967	0.000
Grassland improvement	-0.105	-0.120	-0.090	0.008	-13.770	0.000
Area Fennoscandian species-rich dry-mesic lowland grassland (log x + 1)	0.274	0.261	0.287	0.007	41.513	0.000
Area Semi-natural dry grassland and shrubland on calcareous substrates (log x + 1)	0.139	0.127	0.151	0.006	23.191	0.000

Supplementary Table 7. Reclassification of present-day (2016) terrain map to match the historical dataset. Water in the historical dataset was added as a contemporary layer from the terrain map and therefore exactly matches the modern map. Valuable grasslands from the semi-natural grassland database (http://www.sjv.se/tuva) were added on top of the modern map as open land.

**Historical Map**[1,2]	**Present-day map** [Swedish Terrain Map 2016: https://www.lantmateriet.se/sv/Kartor-och-geografisk-information/Kartor/oppna-data/hamta-oppna-geodata]
Arable	Arable land
	Fruit farm
Open	Other open land
	Group of buildings with courtyard
	High-rise buildings
	Low-rise buildings
	Industrial area
	Leisure homes
	Other open land with isolated trees
Forest	Forest, coniferous and mixed
	Deciduous forest
Water	Water body
	Water body with unclear shoreline

Supplementary Table 8. Fifty-six vascular plant taxa recorded as specialist species in Swedish semi-natural grasslands[3].

Ajuga pyramidalis	*Leontodon hispidus*
Antennaria dioica	*Leucanthemum vulgare*
Armeria maritima	*Linum catharticum*
Arnica montana	*Luzula multiflora*
Bistorta vivipara	*Lychnis flos-cuculi*
Botrychium spp.	*Nardus stricta*
Briza media	*Ophioglossum vulgatum*
Cardamine pratensis	*Orchis mascula*
Carex hostiana	*Parnassia palustris*
Carex panicea	*Pedicularis sylvatica*
Carlina vulgaris	*Pimpinella saxifraga*
Centaurium spp.	*Pinguicula vulgaris*
Cirsium heterophyllum	*Plantago media*
Crepis praemorsa	*Platanthera bifolia*
Dactylorhiza incarnata	*Polygala spp.*
Dactylorhiza maculata	*Primula farinosa*
Danthonia decumbens	*Primula veris*
Dianthus deltoides	*Pulsatilla vulgaris*
Epipactis palustris	*Rhinanthus spp.*
Euphrasia spp.	*Scorzonera humilis*
Filipendula vulgaris	*Succisa pratensis*
Galium verum	*Thymus serpyllum*
Gentiana pneumonanthe	*Trifolium fragiferum*
Gentianella campestris	*Triglochin maritimum*
Gymnadenia conopsea	*Triglochin palustre*
Helianthemum spp.	*Trollius europaeus*
Helictotrichon pratense	*Veronica officinalis*
Hypochaeris maculata	*Veronica spicata*

Supplementary Table 9. Pearson correlation of local and landscape predictor variables in 46 796 Swedish semi-natural grasslands. Non-independence of corresponding past and present landscape variables (**bold blue**) and correlations between forest and both open and arable landscape cover (**bold green**), meant that landscape forest cover was not included in any analysis and arable and open land from only one time period was included in each model explaining grassland specialist richness. Hab. Dir. 1 & 2 refer to the fraction of grassland covered by Fennoscandian species-rich dry-mesic lowland grassland and Semi-natural dry grassland and shrubland on calcareous substrates, respectively.

	Grassl. Area (log)	Grassl. Het.	Grassl. Improv.	Grassl. Open	Hab. Dir. 1	Hab. Dir. 2	Past Landsc. Arable	Present Landsc. Arable	Past Landsc. Open	Present Landsc. Open	Past Landsc. Forest	Present Landsc. Forest	Past Landsc. Het.	Present Landsc. Het.
Grassl. Area (log)	1	0.4	0.07	-0.03	0.32	0.3	0.08	0.11	0.02	0.24	-0.22	-0.3	0.08	0.18
Grassl. Het.	0.4	1	0.16	0.07	0.2	0.2	0.01	0.03	0.04	0.14	-0.09	-0.14	0.03	0.07
Grassl. Improv.	0.07	0.16	1	0.05	-0.03	-0.05	0.13	0.12	-0.06	-0.04	-0.04	-0.04	0.01	0.03
Grassl. Open	-0.03	0.07	0.05	1	-0.02	0.14	0.04	0.06	0.08	0.17	-0.12	-0.15	-0.03	0
Hab. Dir. 1	0.32	0.2	-0.03	-0.02	1	-0.11	0.01	-0.01	-0.09	-0.14	0.07	0.09	0.03	0
Hab. Dir. 2	0.3	0.2	-0.05	0.14	-0.11	1	0.05	0.07	0.25	0.38	-0.25	-0.28	0.01	0.05
Past Landsc. Arable	0.08	0.01	0.13	0.04	0.01	0.05	1	**0.95**	-0.27	0.16	**-0.57**	-0.62	-0.08	0.26
Present Landsc. Arable	0.11	0.03	0.12	0.06	-0.01	0.07	**0.95**	1	-0.25	0.12	-0.56	**-0.67**	-0.12	0.25
Past Landsc. Open	0.02	0.04	-0.06	0.08	-0.09	0.25	-0.27	-0.25	1	**0.33**	**-0.27**	0.1	0.25	-0.15
Present Landsc. Open	0.24	0.14	-0.04	0.17	-0.14	0.38	0.16	0.12	**0.33**	1	-0.34	**-0.57**	0.1	0.32
Past Landsc. Forest	-0.22	-0.09	-0.04	-0.12	0.07	-0.25	**-0.57**	-0.56	**-0.27**	-0.34	1	**0.81**	-0.33	-0.38
Present Landsc. Forest	-0.3	-0.14	-0.04	-0.15	0.09	-0.28	-0.62	**-0.67**	0.1	**-0.57**	**0.81**	1	-0.16	-0.53
Past Landsc. Het.	0.08	0.03	0.01	-0.03	0.03	0.01	-0.08	-0.12	0.25	0.1	-0.33	-0.16	1	**0.67**
Present Landsc. Het.	0.18	0.07	0.03	0	0	0.05	0.26	0.25	-0.15	0.32	-0.38	-0.53	**0.67**	1

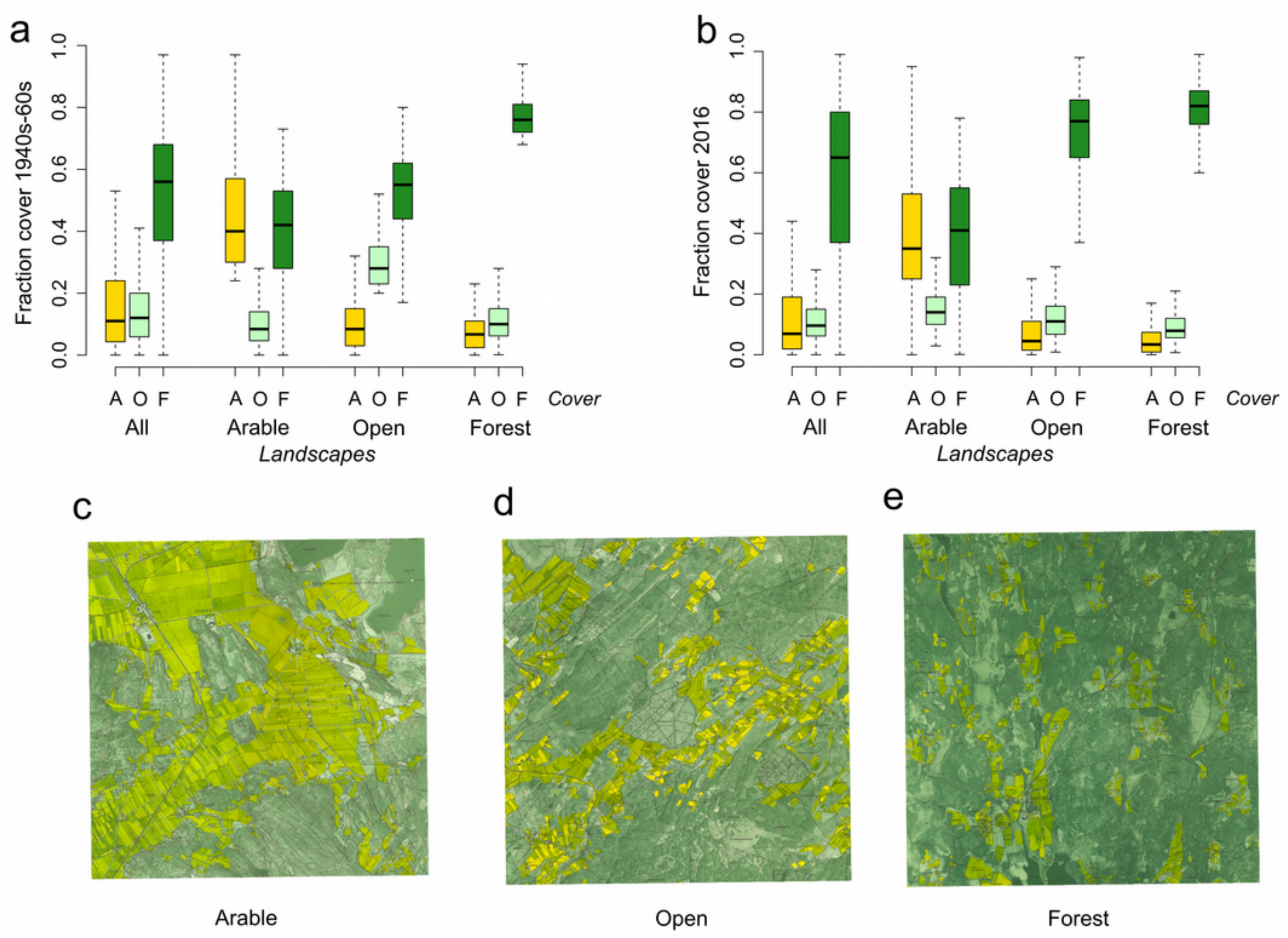

Supplementary Figure 1. Summary information of past and present Swedish landscapes. Fraction cover (median, interquartile range and range excluding outliers defined as quartiles ± 1.5 × the interquartile range) of arable (yellow), open (light green) and forest (dark green) in 1940s-60s [a] and 2016 [b] for all 46 796 landscapes and for arable, open and forest landscapes, defined as the landscapes with the top 25% (n=1683) cover of each land-use category in the historical time period. Panels [c-e] show the landscape with the median cover of arable (yellow), open (light green) and forest (dark green) from each respective landscape type. Map images from Lantmäteriet's Historical Archive

Supplementary Methods

Random forest exploratory analysis.

The Swedish governmental database of valuable semi-natural grasslands (TUVA database – http://www.sjv.se/tuva) contains information on the presence and cover of 30 types of grassland habitat from the EU Habitat Directive (92/43/EEC) within each inventoried grassland, plus categories for "mosaic" and "other". We considered it likely that the presence/amount of some of these habitats would have a positive effect on the number of grassland specialist species recorded in a grassland, but including all 32 did not seem like a sensible approach. In order to establish which grassland types have the strongest links to species richness, we ran a random forest model (using the randomForest function in the randomForest package in R[4,5]) containing number of grassland specialist species in all 46 796 grasslands as the response, with the areas of each of the 32 habitat categories and total grassland area (both in hectares) as explanatory variables. We used the default 500 trees and set the minimum terminal node size to 497 (1% of the total data) for computational efficiency. Based on the large differences in increase in node purity in relation to the remainder of the grassland types (see Supplementary Table 10, below), we chose to include Fennoscandian species-rich dry-mesic lowland grassland and Semi-natural dry grassland and shrubland on calcareous substrates for inclusion in the main analysis.

Supplementary Table 10. Degree by which the inclusion of 32 grassland habitat types increased explanatory power in a random forest model of species richness across grassland patches.

Habitat type	Increase in Node Purity
Fennoscandian species-rich dry-mesic lowland grassland	114642.0676
Grassland area	59556.05999
Semi-natural dry grassland and shrubland on calcareous substrates	49806.13931
Molina meadows on calcareous, peaty or clayey-silt-laden soils	18256.36484
Nordic alvar and precambrian calcareous flatrocks	14081.3205
Species-rich extensively managed hay meadows	11247.60234
Mosaic	9068.706872
Alkaline fens	5741.421328
Species-rich Nardus grassland on silicious substrates in mountain areas	5100.535326
Fennoscandian wooded pastures	4211.358568
Fennoscandian wooded meadows	2519.375989
Cultivated grassland (often ex-arable field now grazed)	1811.680659
Juniper grassland on heath/calc grassland	1771.000579
Boreal Baltic coastal meadows	1117.665739
Rupicolous calcareous or basophilic grasslands of the Alysso-Sedion albi, Alvar	1111.18986
Atlantic salt meadows (Glauco-Puccinellietalia maritimae)	787.7713811
European dry heaths	721.4654355
Siliceous rock with pioneer vegetation	468.6912328
Limestone pavements	178.4061936
Oligotrophic to mesotrophic standing waters	132.8451959
Petrifying springs with tufa formation	122.3724126
Northern Atlantic wet heaths with Erica tetralix	97.5118702
Fixed coastal dunes with herbaceous vegetation	90.95857504
Salicornia and other annuals colonizing mud and sand (glasswort mudflats and sandflats)	80.53968206
Inland dunes with open Corynephorus and Agrostis grasslands	76.61248749
Calcareous fens with Cladium mariscus and species of the Caricion davallianae	56.59538366
Hydrophilous tall herb fringe communities of plains and of the montane to alpine levels	42.4547945
Transition mires and quaking bogs	22.59327521
Xeric sand calcareous grasslands	7.101046492
Other	0
Calcareous rocky slopes with chasmophytic vegetation	0
Large shallow inlets and bays	0
Dry sand heaths with Calluna and Empetrum nigrum	0

Supplementary References

1. Auffret, A. G. *et al.* HistMapR: Rapid digitization of historical land-use maps in R. *Methods Ecol. Evol.* **8,** 1453–1457 (2017).

2. Auffret, A. G. *et al.* Data from: HistMapR: Rapid digitization of historical land-use maps in R. Figshare http://dx.doi.org/10.17045/sthlmuni.4649854 (2017).

3. Swedish Board of Agriculture. *Ängs- och betesmarks- inventeringen 2002–2004*. (Swedish Board of Agriculture, Jönköping, 2005).

4. Liaw, A. & Wiener, M. Classification and regression by randomForest. *R News* **2,** 18–22 (2002).

5. R Development Core Team. *R: A language and environment for statistical computing*. (R Foundation for Statistical Computing, Vienna, 2017).
